# Notes

**Published:** 1900-02

**Authors:** 


### NOTES.

In a call for the establishment of a State Breeders' Association in Pennsylvania the committee issuing the call state, among other facts, that Pennsylvania breeders need a good many things which a strong State organization will help them to secure. They need to learn more about each other and more about their business interests. They need better and cheaper shipping facilities for pure-bred stock. They need better classification of stock at fairs, better premiums, and better judging. And they need a State Fair that will properly represent and encourage the live-stock industry. We note on the first programme a paper by State Veterinarian Pearson on "Sanitary Barns," surely a subject of the deepest and most important interest to the breeders.

The JOURNAL is in receipt of a photographic reproduction of an "osteoma," weighing fifteen pounds and three-quarter ounces, twenty-two inches in circumference, eight inches in diameter, with covering of only one inch thickness, removed from the popliteal region of a six-year-old mare on November 21, 1899, by Dr. Alexander Crowforth, of Lockport, N. Y. This growth was the result of an injury occurring some seven months prior to operative procedure.

Several of Philadelphia's milkmen now announce on their cards and letter-heads the names of the veterinarians who fill the rôle of inspector of the herds that supply their dairies with milk.

*Farmers' Bulletin* No. 708, issued by the United States Department of Agriculture, is one of many that affords valuable information to the farmer. In describing the various salt-bushes and the non-productive soil on which they will grow it also treats of their value as food for the horse, cow, sheep, and other animals. The scope of work done by this department in the general interest of the great body of our people engaged in agriculture is a continued source of wonder.

*Bulletin* No. 61 of the West Virginia Agricultural Experiment Station is an an interesting contribution to successful sheep feeding. The tables are complete in details of the manner in which the experiments were conducted.

Veterinarian John Wende reports the prevalence of rabies in and around Buffalo since November, 1898, causing the loss of many horses, cattle, one deer at the Park Zoo, hogs, dogs, cats, and at least four cases of hydrophobia among members of the human family. An ordinance, to take effect February 26, 1900, requiring all dogs on the street to be led by a leash, has been passed by Buffalo City Councils.

The Governor of Texas recently announced quarantine measures against Colorado and some other Western States, on the grounds that tuberculosis was existing among their cattle, and the live-stock interests of the "Lone Star State" were endangered.

Two thousand and seventy-two entries were made at the Westminster Kennel Club Show in New York in February.

Acton, England, has a "Home of Rest" for horses of cabmen, grocers, etc., maintained by the charitably disposed members of society, where the best of care, medical treatment, etc., are given.

Dr. George M. Walrod, of Storm Lake, Iowa, states that glanders exists in Buena Vista County, Iowa. State Veterinarian Gibson and his assistant, Dr. Hammond, have already destroyed some thirty-four horses. The disease seems to have been introduced through the movement of horses incidental to construction of a railroad through that territory.

